# Application of fluorescein combined with methylene blue in sentinel lymph node biopsy of breast cancer

**DOI:** 10.1038/s41598-021-91641-1

**Published:** 2021-06-09

**Authors:** Liang Li, Ning Gao, Ai Qing Yang, Wen Hao Xu, Yu Ding, Jun Chu, Xiao Na Lin, Jia Qi Liu

**Affiliations:** 1 Department of Breast Surgery, Zibo Central Hospital, Shandong First Medical University, Zibo, Shandong Province China; 2 Zibo Center for Disease Control and Prevention, Zibo, Shandong Province China

**Keywords:** Breast cancer, Cancer therapy, Cancer

## Abstract

Sentinel lymph node biopsy (SLNB) for axillary lymph node staging in early breast cancer has been widely recognized. The combination of radio-colloids and dye method is the best method recognized. The reagents and equipment required in the process of the combined method are complex and expensive, so there are certain restrictions in the use of primary medical institutions. As a new tracer, fluorescent tracer technology has attracted much attention. We aimed to evaluate the feasibility and safety of fluorescein for SLNB in breast cancer. In this study, a total of 123 patients with breast cancer were divided into group A (n = 67) and group B (n = 56). The efficacy of Indocyanine green (ICG) combined with methylene blue (group A) and fluorescein combined with methylene blue (group B) in SLNB of breast cancer was compared, complications were observed at the same time. No local or systemic reactions were observed in the two groups. In group A, Sentinel lymph nodes of breast cancer were detected in 63 patients, with a detection rate of 94.0% (63/67), a false-negative rate of 7.5% (4/53). In group B, Sentinel lymph nodes of breast cancer were detected in 52 patients, with a detection rate of 92.9% (52/56), a false-negative rate of 7.5% (3/40). There was no significant difference in biopsy results between the two groups. This prospective clinical study suggests that SLNB using fluorescein and ultraviolet LED light is feasible in breast cancer patients. No adverse reactions were observed in this study, but larger studies are needed to properly assess the adverse reaction rate.

## Introduction

Sentinel lymph node biopsy (SLNB) for axillary lymph node staging in early breast cancer has been widely recognized. Blue dye, radio-colloids, or both can be used to identify the sentinel lymph node^1,2^. The biggest problem of SLNB using blue dye alone is that its detection rate is only 70–86%. The combination of radio-colloids and the blue dye method can significantly improve the detection rate of SLNB, which is the best method recognized in the clinic^3^. However, Radio-colloids are expensive, complex, require the cooperation of the nuclear medicine department, which is difficult to carry out in primary hospitals. Also, It needs to be injected preoperatively, which can cause significant pain to patients and also cause concern for patients and physicians about radiation exposure.

As a new tracer, fluorescent tracer technology has attracted much attention^4^. Indocyanine green (ICG) is the most commonly used fluorescent tracer^5^. It reflects fluorescence when excited by certain wavelengths of near-infrared light, and the signal is processed by a computer to transmit the image to a screen. Therefore, a fluorescent imaging system with a near-infrared camera is required to perform SLNB by looking at the monitor rather than the surgical field.

Fluorescein is a fluorescent tracer widely used in ophthalmology and optometry. It can be excited by ultraviolet or blue light through thin tissue to appear green and yellow light. SLNB use fluorescein requires an only blue or ultraviolet light torch, which reduces the cost of surgery and is easy to be applied in primary hospitals. Recent animal studies have shown that fluorescein can also be used to locate axillary lymph nodes in rabbits^6^.

In this study, the efficacy of ICG combined with methylene blue and fluorescein combined with methylene blue in SLNB of breast cancer was compared.

## Materials and methods

### Clinical data

From July 2019 to December 2020, 123 patients with feasible SLNB of breast cancer admitted to our hospital were studied. According to the order of admission, they were divided into group A (n = 67) and group B (n = 56). (Table 1) All of them provided written informed consent. The protocol and consent procedures were approved by the Institutional Review Board of Zibo Central Hospital affiliated with Shandong First Medical University, and all experiments were performed following relevant guidelines and regulations. The Chinese Clinical Trial Registry (available at http://www.chictr.org.cn/) approved the clinical nature of this study (registration number: ChiCTR2000036990).

**Table 1:** Clinical characteristic of the patients enrolled in this study.

Characteristic	Value	
	Group A (n = 67)	Group B (n = 56)
Age (years)	47 (26–73)	49 (30–72)
Body mass index (kg/m^2^)	23.3 (18–36)	23.9 (17–37)
Tumor size (cm)	1.8 (0.5–4.0)	1.6 (0.7–3.8)
TNM staging		
**Tumor**		
pT1	46 (68.7%)	39 (69.6%)
pT2	21 (31.3%)	17 (30.4%)
**Node**		
pN0	13 (19.4%)	15 (26.8%)
pN1	49 (73.1%)	34 (60.7%)
pN2	5 (7.5%)	7 (12.5%)

TNM staging, Tumor, node and metastasis staging.

### Methods

First, the fluorescent dye was injected. ICG solution was used in group A (25 mg ICG powder was diluted with 9 mL of water for injection, and then 0.1 mL of the diluted solution was extracted with a 1 mL syringe and continued to be diluted to 1 mL with 0.9% normal saline), and fluorescein solution was used in group B(1:5 fluorescein solution (10% fluorescein solution 1 mL and 0.9% normal saline 4 mL were mixed into the solution, with an average pH value of 8.97 (8.95–9.02)^7^). The intradermal injection was made into the outer upper quadrant of the areola at 3–4 points, the total amount is about 0.1 to 0.3 ml. Group B patients were tested for fluorescein allergy (0.1 ml 10% fluorescein solution was diluted to 5 ml with 0.9% normal saline, injected intravenously, and observed for 10 min) no positive results were found^8^.

After 3 min, a 1 ml syringe was used to select 1–3 points on the outside of the areola for intradermal injection of methylene blue, with a total amount of about 0.1–0.3 ml.

**Figure 1:**
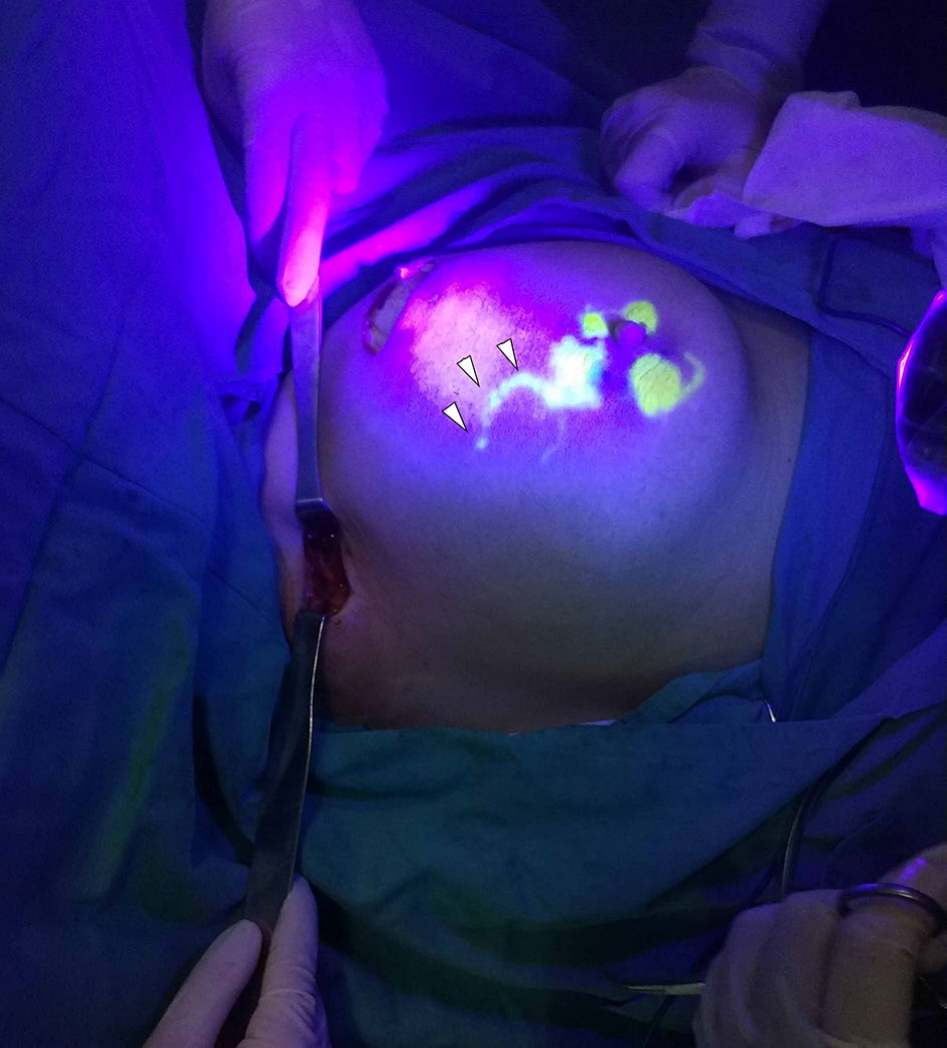
Lymph vessel (arrow) shown by fluorescein during the operation.

10 min later, the operating lights were turned off. The images of group A were collected using the near-infrared camera system, and group B was marked with an ultraviolet (wavelength 395 nm) LED torch (LOFTEK, China) to mark the direction of lymph vessels and the location of their disappearance (Fig. 1).

**Figure 2:**
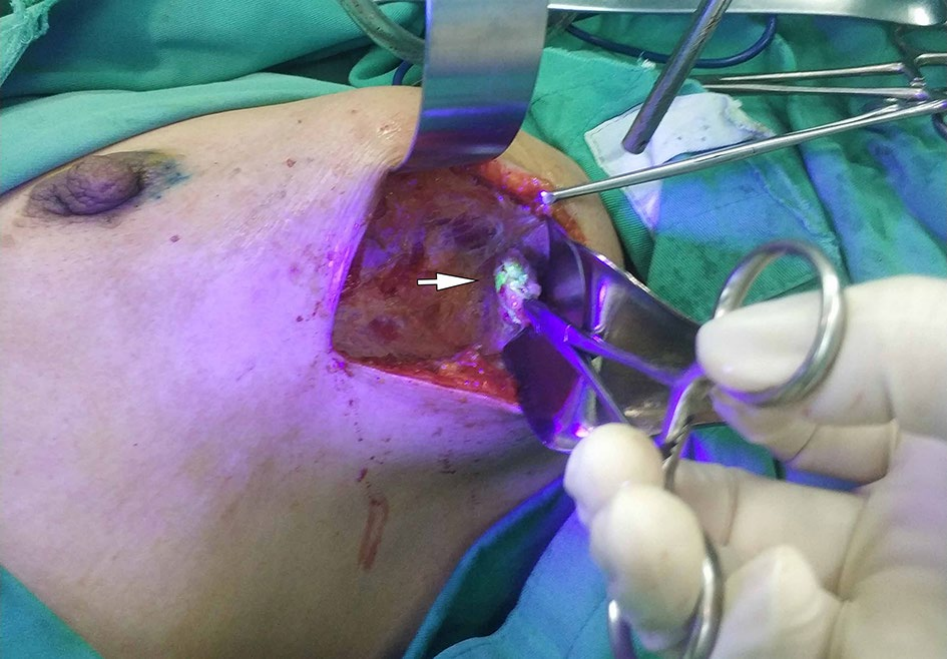
Lymph node (arrow) shown by fluorescein during the operation.

An incision was made approximately 1 cm from the distal end of lymph vessel disappearance. If there is no obvious lymph vessel, an incision is made at the inferior axillary fold. Group A was guided by an infrared probe and group B was guided by the ultraviolet LED torch to identify the fluorescent and methylene blue-stained lymph vessels, and then followed the lymph vessels to remove the sentinel lymph nodes (Fig. 2).

All lymph nodes were examined by frozen pathology and conventional paraffin pathology. For the sentinel lymph nodes with no metastatic cancer, only the lower axillary lymph nodes were dissected. Routine axillary lymph node dissection was performed in patients with sentinel lymph node metastasis.

### Main outcome measures

The results of SLNB in groups A and B were compared, including detection rate, false-negative rate, number of sentinel lymph nodes.

### Evaluation of complications in fluorescein group

Anaphylaxis and systemic urticaria were observed within 20 min of the operation. The skin necrosis at the injection site was observed within 1 week. Liver and kidney functions were measured 7 days after the operation. The color changes of skin and urine at the injection site were observed 2 days after the operation.

### Statistical analysis

All statistics were analyzed using IBM SPSS 22.0 statistics software. (IBM Co., Armonk, NY, USA) Continuous variables are expressed as median or mean, and categorical variables as percentages. T-test was used for the comparison of measurement data and X^2^ test was used for the comparison of counting data. When the p-value was less than 0.05, the difference was considered statistically significant.

## Results

### Comparison of biopsy results between the two groups

A total of 123 eligible women with breast cancer were enrolled (Table 1). The patients ranged in age from 26 to 73 years.

No local or systemic reactions were observed in 67 patients in group A. Sentinel lymph nodes of breast cancer were detected in 63 patients, with a detection rate of 94.0% (63/67), a false-negative rate of 7.5% (4/53).

Group B was no local or systemic adverse reactions in 56 patients. Sentinel lymph nodes of breast cancer were detected in 52 patients, with a detection rate of 92.9% (52/56), a false-negative rate of 7.5% (3/40). There was no significant difference in biopsy results between the two groups (Table 2).

**Table 2:** Comparison of biopsy results between the two groups.

	Group A (n = 67)			Group B (n = 56)			P
	ICG + MB	(ICG	MB)	Flu + MB	(Flu	MB)	(ICG + MB vs Flu + MB)
Detection Rate (%)	94.0 (63/67)	89.6 (60/67)	79.1 (53/67)	92.9 (52/56)	87.5 (49/56)	83.9 (47/56)	1.000
False-negative rate (%)	7.5 (4/53)	–	–	7.5 (3/40)	–	–	1.000
Number of SLNs	3.0 (1–6)	3.0 (1–5)	3.0 (1–6)	3.5 (1–6)	3.0 (1–6)	3.0 (1–5)	0.406

*ICG* Indocyanine green, *MB* Methylene blue, *Flu* Fluorescein, *SLNs* sentinel lymph nodes.

### Sentinel lymph node biopsy details

The median time from the injection of fluorescein and ICG to the initial incision was 35 and 14 min, respectively, and the median time from the initial incision to the end of the sentinel node biopsy was 15 min with the ultraviolet LED light. There was no significant difference in the median number of sentinel lymph nodes detected between the two methods (3.0 VS 3.5, p = 0.406) . Sentinel lymph nodes were detected in 52 of the 56 patients in group B, and the detection rate was no different from that in group A (92.9% VS 94.0%, P = 1.000). In group A, SLNB found metastasis in 49 cases, axillary lymph node dissection found metastasis in 53 cases, with a false-negative rate of 7.5% (4/53).In group B, SLNB found metastasis in 37 cases, axillary lymph node dissection found metastasis in 41 cases. Among them, 1 case in the fluorescein group was unsuccessful in SLNB, and the dissected axillary lymph node had metastasis, with a false-negative rate of 7.5% (3/40), and there was no significant difference between the two groups (P = 1.000).

### Complications

None of the patients showed anaphylaxis or systemic urticaria after intradermal injection of fluorescein, and no skin necrosis was observed at the injection site. Serum creatinine and liver function were in the normal range on day 1, day 8, and month 1. All patients' urine glowed yellow within 10 min of fluorescein injection, but for no more than a day. Skin discoloration also disappears within a day.

## Discussion

The detection rate of the fluorescein combined with methylene blue group was 92.9%, and sentinel lymph nodes were visible under ultraviolet LED light, which was easy to be detected in most patients. Sentinel lymph node biopsy was unsuccessful in 4 cases, and axillary lymph node metastasis was found in 1 case. In the early stages of this study, it was difficult to detect sentinel lymph node using fluorescein in patients with relatively high body mass index. It is well known that weight is a predictor of failure to identify sentinel lymph node, however, once researchers were familiar with the use of fluorescein and ultraviolet LED light, BMI did not affect SLNB.

Studies on SLNB with ICG showed that this reagent was a feasible lymph node localization agent with a high detection rate and low false-negative rate^9^. However, because ICG emits light at a wavelength of 830 nm (nm), this is considered near-infrared, A fluorescence imaging system with a near-infrared camera is required, and SLNB should be performed by looking at the monitor rather than the field^10–12^. In contrast, fluorescein emits light with a wavelength of 512 nm, which is in the visible spectrum. Thus, surgeons can perform SLNB by visually observing the operating area without using special equipment.

The injected fluorescein is delivered to the liver and rapidly converted into fluorescein glucuronic acid, which is then excreted in the urine at a 1.75 mL /min/kg renal clearance rate^13^. Discoloration of the urine was observed in all patients injected with fluorescein, but this did not last for more than 24 h. There was no abnormal creatinine or liver function, no allergic reaction, or general urticaria. The commonly used intravenous dose of fluorescein for retinal angiography is 250–500 mg^14^. In this study, however, only 80 mg of fluorescein (4 ml 1:5 diluted 10% fluorescein) was intradermally injected for SLNB in each patient, and no systemic complications. The mean pH of 1:5 diluted 10%fluorescein was 8.96. Because it is weakly alkaline, the injection site was carefully monitored due to the intradermal injection. However, no local complications such as skin necrosis, pain, or burning were observed in any of the patients.

However, our study had several limitations. First, no patients detected sentinel lymph node in the inner breast quadrant with this drug. Therefore, the feasibility of SLNB in the inner breast quadrant with fluorescein and ultraviolet LED cannot be evaluated. Therefore, further evaluation of larger sample size is needed. Second, fluorescein emits enough light to penetrate thin tissue, but not the thick tissue that includes underarm skin and subcutaneous fat. Therefore, the exact location of the SLN cannot be observed until the skin is cut open. Besides, if techniques using fluorescein are further optimized and developed, for example, to monitor the signal intensity of reflected light in real-time, then unnecessary removal of worthless lymph nodes as sentinel lymph nodes can be avoided.

Despite the limitations, this prospective clinical study suggests that SLNB using fluorescein and ultraviolet LED light is feasible in breast cancer patients. Since the technology uses light in the visible spectrum, it is easy to use and economical, etc., if the results of further research also prove the effectiveness of the technology, it will be likely to be widely used. At the same time, no adverse reactions were observed in this study, but larger studies are needed to properly assess the adverse reaction rate.
